# A Comparative Study of Propofol-Dexmedetomidine Versus Propofol-Ketamine for the Anesthetic Management of Patients During Endoscopic Retrograde Cholangiopancreatography

**DOI:** 10.7759/cureus.74472

**Published:** 2024-11-26

**Authors:** Ipshita Garg, Shahbaz Hasnain

**Affiliations:** 1 Anaesthesiology, Dr. D.Y. Patil Medical College, Hospital and Research Centre, Dr. D.Y. Patil University (Deemed to be University), Pune, IND

**Keywords:** dexmedetomidine, endoscopic retrograde cholangiopancreatography (ercp), hemodynamic stability, ketamine, propofol, recovery time, sedation, total intravenous anaesthesia

## Abstract

Introduction

Endoscopic retrograde cholangiopancreatography (ERCP) requires effective sedation for patient comfort and procedural success. This study compares propofol-dexmedetomidine (group DP) and propofol-ketamine (group KP) for anesthetic management during ERCP.

Methods

This randomized, double-blinded study enrolled 50 patients (aged 18-60 years) scheduled for ERCP at Dr. D.Y. Patil Medical College and Research Centre, Pune, Maharashtra, India. Patients were randomly allocated to group DP (n=25) or group KP (n=25). Hemodynamic parameters, propofol consumption, recovery time, post-procedural pain (visual analog scale [VAS]), sedation levels (Ramsay sedation score), and post-operative side effects were recorded and analyzed.

Results

Group DP showed significantly lower heart rates, systolic blood pressure, and mean arterial pressure throughout the procedure (p<0.001). Group KP had lower post-operative VAS scores only in the initial post-operative period (p<0.001 at 15 minutes) but similar Ramsay sedation scores as compared to group DP. Total propofol consumption was lower in group DP, though not statistically significant (378.9 vs 454.8 mg, p=0.08). Group DP required significantly less additional propofol bolus (7.8 vs 18 mg, p<0.001) and had shorter recovery times (7.24 vs 15.2 minutes, p<0.001). Group DP showed no incidence of post-operative nausea and vomiting (PONV) or post-operative cognitive dysfunction (POCD), while group KP had a 26.7% incidence of PONV and a 66.7% incidence of POCD.

Conclusion

The propofol-dexmedetomidine combination offers more stable hemodynamic control, lower propofol requirements, and quicker recovery times for ERCP anesthesia. It is recommended for patients requiring tight cardiovascular control and rapid post-procedure recovery.

## Introduction

Endoscopic retrograde cholangiopancreatography (ERCP) has emerged as a vital diagnostic and therapeutic procedure in the management of biliary and pancreatic diseases [1]. While less invasive than traditional surgical approaches, ERCP presents unique challenges for anesthesiologists, necessitating careful consideration of anesthetic techniques to ensure patient comfort, procedural success, and minimization of complications [2].

The ideal anesthetic regimen for ERCP should provide adequate muscle relaxation, analgesia, and amnesia while maintaining hemodynamic stability and allowing for rapid recovery [3]. Propofol, a short-acting intravenous anesthetic, has gained popularity for sedation during ERCP due to its rapid onset and offset of action [4]. However, propofol alone may not provide sufficient analgesia and can lead to dose-dependent respiratory depression [5].

To optimize anesthetic management during ERCP, various drug combinations have been explored. Two promising approaches involve combining propofol with either dexmedetomidine or ketamine [6,7]. Dexmedetomidine, a highly selective α2-adrenergic agonist, offers sedative, anxiolytic, and analgesic properties without significant respiratory depression [8]. Ketamine, an N-methyl-D-aspartate (NMDA) receptor antagonist, provides dissociative anesthesia and analgesia while preserving respiratory drive and airway reflexes [9].

This comparative study aims to evaluate the efficacy and safety of propofol-dexmedetomidine versus propofol-ketamine combinations for anesthetic management during ERCP procedures. By assessing parameters such as hemodynamic stability, propofol consumption, recovery times, post-procedural pain levels, and sedation scores, we seek to determine which combination offers the most favorable anesthetic profile for ERCP patients.

The findings of this study may contribute to the development of optimized anesthetic protocols for ERCP, potentially improving patient outcomes, procedural success rates, and post-operative recovery while minimizing anesthesia-related complications.

## Materials and methods

This randomized, double-blinded comparative study was conducted at Dr. D.Y. Patil Medical College and Research Centre, Pune, Maharashtra, India, over a period of 1.5 years. The study protocol was approved by the Institutional Ethics Committee (IESC/PGS/2022/142). It was stated in the approval certificate that the synopsis titled "A Comparative Study of Patients During Endoscopic Retrograde Cholangiopancreatography (ERCP)" is ethically approved. Written informed consent was obtained from all participants prior to enrollment.

The parameter taken for the estimation of the sample size was recovery time from the study conducted by Abdalla et al. published in the Egyptian Journal of Anaesthesia in 2015 using WinPepi software [10]. The study population consisted of 50 patients aged 18-60 years who were scheduled for ERCP. Inclusion criteria encompassed American Society of Anesthesiologists (ASA) physical status I, II, and III patients. Exclusion criteria included patients with severe cardiac, respiratory, hepatic, or renal dysfunction, history of allergies to study medications, pregnancy, and inability to provide informed consent. Patients were randomly allocated into two groups of 25 each using a computer-based randomization method: group DP (propofol-dexmedetomidine) and group KP (propofol-ketamine).

Double-blinding was ensured. Prior to the procedure, all patients underwent a thorough pre-anesthetic evaluation, including a detailed medical history, physical examination, and review of laboratory investigations. Standard fasting guidelines were followed. On arrival in the endoscopy suite, intravenous access was established, and standard monitoring was initiated, including electrocardiography, non-invasive blood pressure (BP) measurement, and pulse oximetry.

Group DP received IV dexmedetomidine (1 μg/kg) as a bolus loading dose over 15 minutes. Group KP received IV ketamine (1 mg/kg) as a bolus loading dose over 15 minutes. Premedication, consisting of glycopyrrolate (0.004 mg/kg), midazolam (0.02 mg/kg), and fentanyl (1 μg/kg), was administered at the time of induction.

After preoxygenation, anesthesia was induced using propofol (2 mg/kg IV) and succinylcholine (2 mg/kg IV). The trachea was intubated with an appropriately sized cuffed endotracheal tube under direct laryngoscopy. A loading dose of atracurium (0.5 mg/kg) was administered as a muscle relaxant. Anesthesia was maintained using oxygen and air (50:50) with a closed circuit at a total fresh gas flow of 2 L/min. Maintenance infusions were started. Group DP received IV dexmedetomidine at 0.5 μg/kg/hour and IV propofol infusion at 100μg/kg/min, while group KP received IV ketamine at 0.5 mg/kg/hour and IV propofol infusion at 100 μg/kg/min.

Propofol infusion was titrated, and additional propofol boluses were administered based on the patient’s hemodynamic parameters (20% increase in heart rate [HR] and mean arterial pressure [MAP] above baseline values) and the time of muscle relaxant top-ups. Atracurium top-up doses (0.1 mg/kg) were given every 20 minutes. If the top-ups were given recently and still the hemodynamic parameters would not settle, additional propofol bolus dose (10-20mg) administration would be considered. All procedures were performed with the patients in the prone position. Infusions were discontinued prior to the end of the surgical procedure, considering the patient's vitals and the remaining duration of the procedure.

At the end of the procedure, all infusion drugs were stopped, and neuromuscular block was reversed using neostigmine (0.05 mg/kg) and glycopyrrolate (0.008 mg/kg). Suctioning of secretions was performed, and extubation was performed once extubation criteria were met (patient was conscious, hemodynamically stable, and spontaneously breathing, and had oxygen saturation >95% on spontaneous ventilation mode). Patients were then transferred to the recovery room.

Baseline BP was recorded for all patients. BP was measured every 5 minutes for the first 15 minutes after starting the loading dose. A reading was taken immediately after intubation, followed by BP measurements every 10 minutes throughout the procedure. Additional readings were taken at the stoppage of the hypotensive agent (dexmedetomidine [with ongoing rate of 0.5 μg/kg/hour] and ketamine [with ongoing rate of 0.5 mg/kg/hour] infusion] T(S) and at the time of extubation T(E). MAP for all the BP readings was taken into consideration and documented for all the readings. Recovery time was calculated from the time of extubation to the time of spontaneous eye opening.

Post-procedural pain was assessed using the visual analog scale (VAS) at 15, 30, 45, and 60 minutes post-procedure. Sedation levels were evaluated using the Ramsay sedation score at the same intervals.

Statistical analysis was performed using WinPepi software, with a p-value of <0.05 considered statistically significant for all comparisons.

## Results

The study compared two anesthetic regimens for ERCP procedures: propofol-dexmedetomidine (group DP) and propofol-ketamine (group KP). Demographic profile characteristics were comparable among study groups, as shown in Table 1.

**Table 1:** Baseline characteristics among study groups P-value stands for probability and measures how likely it is that any observed difference between groups is due to chance. Statistical analysis was conducted using the independent t-test. A p-value of less than 0.05 was considered statistically significant. ASA, American Society of Anesthesiologists; DP, dexmedetomidine-propofol, KP, ketamine-propofol

		Group DP (n=25)	Group KP (n=25)	p-Value
Age		49.7±17.8	47.08±16.8	0.59
Weight		61.9±10.1	62.8±8.5	0.74
Gender	Males	15 (60%)	12 (48%)	0.395
	Females	10 (40%)	13 (52%)	
ASA	I	7 (28%)	12 (48%)	0.26
	II	15 (60%)	12 (48%)	
	III	3 (12%)	1 (4%)	

Hemodynamic stability

Group DP demonstrated better hemodynamic control throughout the procedure. HRs were significantly lower in GROUP DP at multiple time points compared to group KP, as shown in Table 2. Similarly, MAPs were generally lower in group DP, as shown in Table 3, indicating more stable cardiovascular parameters in group DP.

**Table 2:** Comparison of heart rate between the two groups Statistical analysis was conducted using the independent t-test. A p-value of less than 0.05 was considered statistically significant. SD, standard deviation; DP, dexmedetomidine-propofol; KP, ketamine-propofol

Heart Rate (Mean±SD)	Group DP (n=25)	Group KP (n=25)	p-Value
Baseline (N=50)	97.8±12.7	94.96±13.9	0.448
After loading dose (N=50)	96.8±12.1	94.96±12.7	0.602
5 minutes (N=50)	92.2±13.8	96.2±15.2	0.331
10 minutes (N=50)	88.2±11.74	97.5±15.8	0.022
15 minutes (N=50)	87.6±11.2	96.4±13.8	0.018
Immediately after intubation (N=50)	93.7±12.7	103.3±15.7	0.022
10 minutes after intubation (N=50)	89.4±12.2	100.5±12.6	0.003
20 minutes after intubation (N=50)	81.6±8.1	99.6±13.9	<0.001
30 minutes after intubation (N=50)	77.9±11.4	99.7±14.9	<0.001
40 minutes after intubation (N=50)	77.4±8.4	99.6±15.1	<0.001
50 minutes after intubation (N=48)	78.8±8.02	96.3±12.7	<0.001
60 minutes after intubation (N=46)	77.5±7.4	94.9±10.6	<0.001
70 minutes after intubation (N=38)	80±9.6	94.9±13.7	<0.001
80 minutes after intubation (N=36)	77.7±9.3	94±15.4	<0.001
90 minutes after intubation (N=32)	78.2±6.7	98.4±13.5	<0.001
100 minutes after intubation (N=22)	83.7±14.4	95.6±19.9	0.126
110 minutes after intubation (N=11)	74.2±5.02	99.5±22.7	0.038
120 minutes after intubation (N=7)	77±6.9	110.7±21.9	0.031
Withdrawal of hypotensive agent (N=50)	82.6±11.6	95.04±13.7	0.001
Time of extubation (N=50)	95.04±8.5	102.4±11.03	0.011
Post-operative 15 minutes (N=50)	79.6±7.1	94.2±10.9	<0.001
Post-operative 30 minutes (N=50)	76.96±6	92.9±11.9	<0.001
Post-operative 45 minutes (N=50)	75.04±6.2	91.8±12.2	<0.001
Post-operative 60 minutes (N=50)	76.6±7.4	90.6±9.9	<0.001

**Table 3:** Comparison of mean arterial pressure among the two groups Statistical analysis was conducted using the independent t-test. A p-value of less than 0.05 was considered statistically significant. SD, standard deviation; DP, dexmedetomidine-propofol; KP, ketamine-propofol

MAP (Mean±SD)	Group DP (n=25)	Group KP (n=25)	p-Value
Baseline	102.6±13.5	99.2±14.2	0.589
After loading dose	85.1±9.1	91.6±14.6	0.09
5 minutes	80.1±8.3	86.6±18.6	0.097
10 minutes	74.2±8.8	86.04±12.9	<0.001
15 minutes	71.04±7.7	89.8±12.9	<0.001
Immediately after intubation	79.6±10.6	93.04±11.2	<0.001
10 minutes after intubation	82.5±11.7	93.6±11.6	<0.001
20 minutes after intubation	79.3±10.9	87.6±19.5	<0.001
30 minutes after intubation	83.5±10.98	90.04±13.3	<0.001
40 minutes after intubation	77.7±14.7	94.4±11.9	<0.001
50 minutes after intubation	68.5±12.1	90.6±11.98	<0.001
60 minutes after intubation	71.78±13.2	87.2±8.4	<0.001

Post-operative pain

Interestingly, group DP had higher initial post-operative VAS scores for pain at 15 minutes (p<0.001), but the scores were similar between groups at later time points, as shown in Table 4.

**Table 4:** Comparison of outcomes in two groups Statistical analysis was conducted using the independent t-test. A p-value of less than 0.05 was considered statistically significant. DP, dexmedetomidine-propofol; KP, ketamine-propofol

Outcomes		Group DP (n=25)	Group KP (n=25)	p-Value
Post-operative VAS (Mean±SD)	15 minutes	0.84±0.8	0.16±0.37	<0.001
	30 minutes	0.3±0.64	0.1±0.27	0.15
	45 minutes	0.6±0.76	0.3±0.27	0.06
	60 minutes	0.28±0.45	0.08±0.27	0.06
Propofol consumption (Mean±SD)	Total consumption	378.9±111.9	454.8±169.9	0.08
	Additional propofol bolus	7.8±4.8	18±5.6	<0.001
Recovery time		7.24±1.5	15.2±1.5	<0.001

Propofol consumption

Although the total propofol consumption was not statistically significant between the groups, group DP required significantly less additional propofol bolus (7.8 mg vs 18 mg, p<0.001), as shown in Table 4. This suggests that the dexmedetomidine combination may allow for reduced propofol requirements.

Recovery time

Recovery time was calculated from time of extubation to the time of spontaneous eye opening. Spontaneous eye opening was considered to be the clinic end point for recovery time. Patients in Group DP had significantly shorter recovery times compared to Group KP (7.24 minutes vs 15.2 minutes, p<0.001), as shown in Table 4. This faster recovery could be advantageous in terms of patient turnover and post-procedure care.

## Discussion

The study compared propofol-dexmedetomidine (group DP) and propofol-ketamine (group KP) for ERCP anesthesia, revealing significant differences in patient outcomes. Demographic factors were similar between groups, aligning with previous studies [11-14]. Hemodynamically, group DP showed better stability with lower HRs and MAPs, consistent with dexmedetomidine's sympatholytic effects [15-17]. This contrasts with ketamine's sympathomimetic action in group KP [18]. Pain management differed, with group KP showing lower immediate post-operative pain scores, reflecting ketamine's potent analgesic properties [18]. However, sedation levels were comparable between groups [19].

Notably, group DP required less propofol, particularly in additional boluses, supporting findings that dexmedetomidine reduces propofol needs [20]. Recovery times were significantly shorter in group DP, aligning with dexmedetomidine's pharmacokinetic profile [21].

The significance of this study lies in its potential to revolutionize the anesthetic management of patients undergoing ERCP. By demonstrating that the propofol-dexmedetomidine combination offers superior hemodynamic stability, reduced anesthetic consumption, and faster recovery times compared to the propofol ketamine regimen, this research underscores a pivotal advancement in procedural sedation. The ability of the propofol-dexmedetomidine combination to provide tighter cardiovascular control and enhance patient safety makes it an ideal choice for individuals with comorbid conditions, reducing intraoperative and postoperative complications. Additionally, the significant reduction in total propofol use not only minimizes the risk of side effects but also presents a cost-effective solution for healthcare providers. These findings highlight the propofol-dexmedetomidine combination as a more efficient, safer, and economically advantageous approach for ERCP sedation, with the potential to improve patient outcomes and procedural success in clinical practice. The propofol-dexmedetomidine combination demonstrated superior hemodynamic stability, reduced propofol requirements, and faster recovery times. These findings support its use for enhanced procedural safety and efficacy, especially in patients requiring stable cardiovascular control and rapid recovery.

The study's implications extend to resource allocation, staff workload, and ERAS (Enhanced Recovery After Surgery) protocol implementation. However, limitations include the sample size and single-center design, suggesting the need for larger, multi-center studies.

## Conclusions

In conclusion, while both combinations provided effective sedation and analgesia, the propofol-dexmedetomidine regimen appears to be preferable for ERCP anesthesia due to its favorable hemodynamic profile, reduced propofol needs, and faster recovery. The propofol-ketamine combination may still have a role where post-procedural pain is a primary concern. Future research should focus on refining dosing protocols, such as the optimal infusion rates and multimodal pain control strategies that explore synergies with ERAS pathways, and investigating long-term outcomes in various endoscopic procedures.
